# Hippo-Independent Regulation of Yki/Yap/Taz: A Non-canonical View

**DOI:** 10.3389/fcell.2021.658481

**Published:** 2021-04-01

**Authors:** Yong Suk Cho, Jin Jiang

**Affiliations:** ^1^Department of Molecular Biology, UT Southwestern Medical Center, Dallas, TX, United States; ^2^Department of Pharmacology, UT Southwestern Medical Center, Dallas, TX, United States

**Keywords:** Hippo, Yap, Taz, CDK7, PRP4K, DCAF12, phosphorylation, ubiquitination

## Abstract

Initially identified in *Drosophila*, the Hippo signaling pathway has emerged as an evolutionarily conserved tumor suppressor pathway that controls tissue growth and organ size by simultaneously inhibiting cell proliferation and promoting cell death. Deregulation of Hippo pathway activity has been implicated in a wide range of human cancers. The core Hippo pathway consists of a kinase cascade: an upstream kinase Hippo (Hpo)/MST1/2 phosphorylates and activates a downstream kinase Warts (Wts)/Lats1/2, leading to phosphorylation and inactivation of a transcriptional coactivator Yki/YAP/Taz. Many upstream signals, including cell adhesion, polarity, mechanical stress, and soluble factors, regulate Hippo signaling through the kinase cascade, leading to change in the cytoplasmic/nuclear localization of Yki/YAP/Taz. However, recent studies have uncovered other mechanisms that regulate Yki/YAP/Taz subcellular localization, stability, and activity independent of the Hpo kinase cascade. These mechanisms provide additional layers of pathway regulation, nodes for pathway crosstalk, and opportunities for pathway intervention in cancer treatment and regenerative medicine.

## Introduction

The regulation of cell growth, proliferation, and cell death is tightly controlled during embryonic development and adult tissue homeostasis not only by environmental cues such as morphogens, cytokines, hormonal signals, and nutrients but also by cell-intrinsic mechanisms. The Hippo signaling pathway, which was initially identified in *Drosophila*, has emerged as an evolutionarily conserved tumor suppressor pathway that regulates tissue growth and organ size in a wide range of species ranging from insects to humans (Pan, 2007; Zhang et al., 2009; Halder and Johnson, 2011). Deregulation of Hippo pathway activity has been implicated in many types of human cancer and other diseases (Yu et al., 2015; Zanconato et al., 2016; Zheng and Pan, 2019). Due to its critical role in developmental biology and human health, the Hippo pathway has been extensively studied over the past decade or so. Through genetic screen in *Drosophila* and RNAi screen in mammalian cells as well as proteomic and bioinformatic approaches, numerous pathway components have been identified that link the Hippo signaling to many upstream regulators and other signaling pathways. The rapid progress in the Hippo signaling field is also reflected by the numerous reviews on this topic. In this review, we focus on recent studies that reveal mechanisms that act in parallel to or downstream of the core Hippo signaling pathway to modulate pathway outputs. We discuss how these findings inform us about new strategies for cancer treatment and regenerative medicine.

## Overview of the Canonical Hippo Signaling Pathway

**FIGURE 1:**
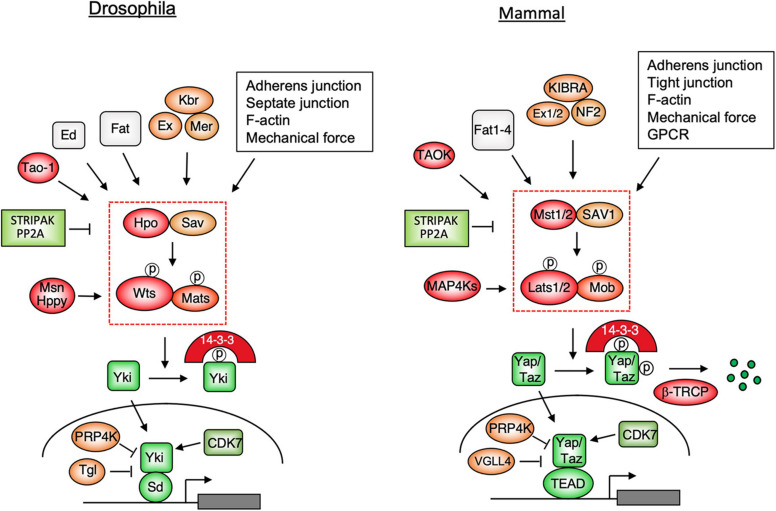
Hippo signaling pathways in *Drosophila* and mammals. Multiple upstream signals act through the Hpo/MST1/2-Wts/Lats1/2 kinase cascade to control the subcellular localization and stability (for mammalian pathway only) of the pathway effectors Yki/Yap/Taz. When phosphorylation of Yki/Yap/Taz by the Hpo kinase cascade is compromised, these pathway effectors enter the nucleus and binds the transcription factors Sd/TEAD1-4 to regulate genes involved in cell growth, proliferation, survival, plasticity, and metabolism.

The core Hippo signaling pathway (Figure 1) contains a kinase cassette: an upstream Ste20 family kinase Hippo (Hpo)/MST1/2, which exists in a complex with Sav/SAV1, phosphorylates and activates a downstream kinase Warts (Wts)/Lats1/2 that forms a complex with Mats/Mob (Harvey et al., 2003; Jia et al., 2003; Pantalacci et al., 2003; Udan et al., 2003; Wu et al., 2003; Lai et al., 2005). Activated Wts/Lats1/2 in turn phosphorylates the Hippo pathway effector Yorkie (Yki) in *Drosophila* and Yes-associated protein (Yap)/Transcriptional activator with PDZ-binding motif (Taz) in mammals, resulting in its cytoplasmic retention by binding to the 14-3-3 protein (Huang et al., 2005; Dong et al., 2007; Zhao et al., 2007; Oh and Irvine, 2008; Zhang et al., 2008; Ren et al., 2010). When Wts/Lats1/2-mediated phosphorylation is compromised, Yki/Yap/Taz translocates into the nucleus where it binds to the Hippo pathway transcription factors Scalloped (Sd)/TEAD1-4 to regulate genes involved in the control of cell growth, proliferation, survival, and metabolism (Wu et al., 2008; Zhang et al., 2008; Zhao et al., 2008; Koo and Guan, 2018; Moya and Halder, 2018; Totaro et al., 2018). In the absence of nuclear Yki/Yap/Taz, Sd/TEAD binds Tgl/VGLL4 and functions as a default pathway inhibitor (Koontz et al., 2013; Jiao et al., 2014).

In several contexts, MAP4K family members Misshapen (Msn)/Happy hour(Hppy)/MAP4Ks act partial-redundantly with Hpo/MST12 to regulate Wts/Lats1/2 (Li et al., 2014, 2015; Meng et al., 2015; Zheng et al., 2015). Interestingly, in *Drosophila* adult intestine, Msn substitutes the function of Hpo and acts as a major upstream kinase for Wts in enteroblasts to regulate stem cell proliferation (Li et al., 2014). Hpo/MST1/2 and Msn are phosphorylated and activated by a conserved kinase Tao-1/TAOK that is recruited to Ex by Schip1 (Boggiano et al., 2011; Poon et al., 2011; Chung et al., 2016). A recent study revealed that enteroblasts in *Drosophila* adult intestine sense the mechanical force generated by food congestion to modulate the Hippo signaling and stem cell activity by regulating the membrane association of Msn and its phosphorylation by Tao-1 (Jiang, 2018; Li et al., 2018). Hpo/MST12 and MAP4Ks are negatively regulated by a large protein complex called STRIPAK that brings PP2A to dephosphorylate and inhibit these kinases (Bae et al., 2017; Zheng et al., 2017; Kim J.W. et al., 2020; Seo et al., 2020; Tang et al., 2020). A recent study suggests that the STRIPAK complex integrates multiple upstream signals to regulate Hippo signaling pathway (Chen et al., 2019).

Genetic studies in *Drosophila* have identified many upstream components that appear to play conserved roles in the Hippo signaling pathway, including the atypical protocadherin family members Fat/Fat1-4 and its binding partner Dachsous (Ds), the FERM domain-containing proteins Expanded (Ex)/Merlin (Mer)/NF2, Kibra (Kbr)/KIBRA, and Spectrin (Figure 1; Fulford et al., 2018; Misra and Irvine, 2018). A genetic modifier screen also identified a cell adhesion molecule called echinoid (Ed) that plays a unique role in *Drosophila* Hippo pathway by recruiting Hpo/Sav to the adherens junction (Yue et al., 2012). The Hippo pathway is also regulated by apical basal polarity complexes, cell junctions including adherens junction, tight junction (mammalian Hippo pathway only), mechanical signals, and soluble factors that activate GPCR pathway (Figure 1; Yu et al., 2015; Fulford et al., 2018). In most of the cases, these upstream regulators act either directly or indirectly to modulate the activity of the Hippo kinase cascade, leading to altered cytoplasmic/nuclear partitioning of Yki/Yap/Taz.

## Hpo Kinase Cascade-Independent Regulation of Yki/Yap/Taz

Although most upstream signals regulate Hippo signaling through modulating the Hpo kinase cascade-mediated phosphorylation of Yki/Yap/Taz, mechanisms that regulate Yki/Yap/Taz activity independent of the core Hippo pathway do exist. For example, an early study indicated that direct interaction between Yki and Ex can sequester Yki in the cytoplasm in *Drosophila* (Badouel et al., 2009). Likewise, direct interaction of Yap with Angiomotin (Amot) also traps Yap in the cytoplasm of mammalian cells (Zhao et al., 2011). Furthermore, Tyr phosphorylation of Yap by Src family kinases can regulate Yap nuclear localization, stability, and activity (Rosenbluh et al., 2012; Taniguchi et al., 2015; Li et al., 2016). In this review, we focus on recent studies that uncover additional mechanisms that control Yki/Yap/Taz activity independent of the Hpo kinase cascade.

## Regulation of Yki/Yap/Taz by Other Cytoplasmic Ser/Thr Kinases

**FIGURE 2:**
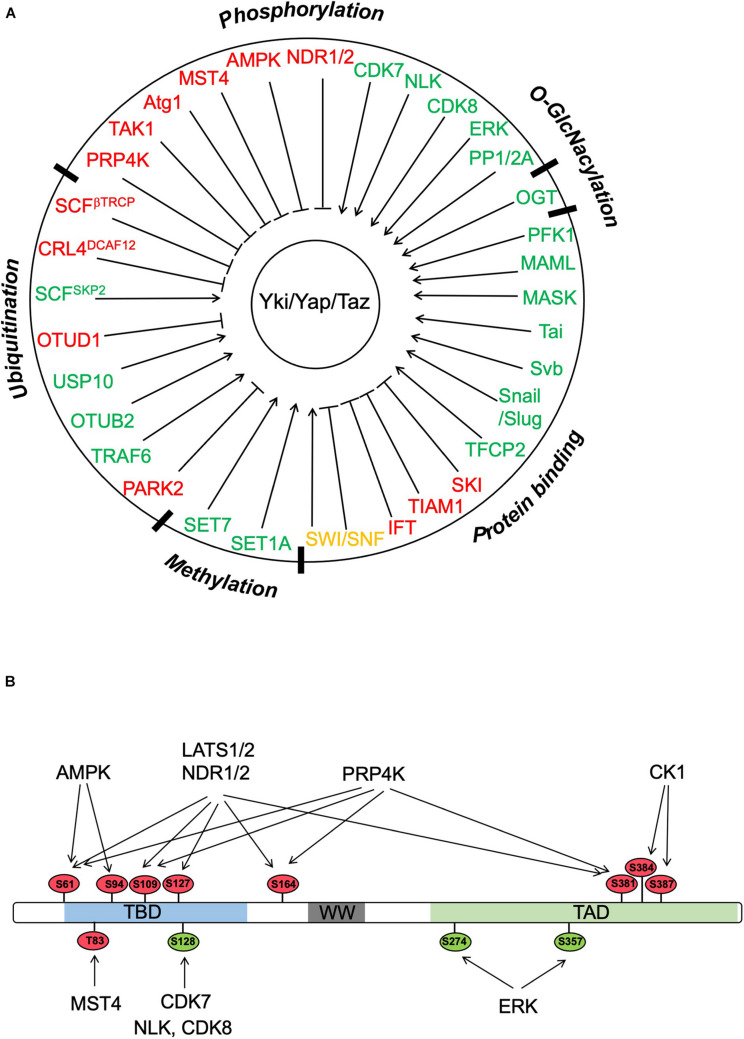
Regulation of Yki/Yap/Taz by PTMs and protein–protein interactions. **(A)** Yki/Yap/Taz can be regulated by multiple posttranslational modifications (PTMs) including phosphorylation, ubiquitination, methylation, and O-GlcNacylation as well as by interacting partners. “Red” and “green” color codes indicate negative and positive regulators of Yki/Yap/Taz, respectively. Of note, SWI/SNF is color coded in “orange” because both negative and positive roles have been implicated depending on the context. **(B)** Yki/Yap/Taz activity can be regulated by multiple Ser/Thr kinases through phosphorylation of the indicated sites (based on Yap1). The phosphorylation sites are color coded with “red” and “green” indicating negative and positive effects on Yap activity, respectively.

Although Wts/Lats1/2-mediated phosphorylation of Yki/Yap/Taz provides a major mechanism that regulates the Hippo pathway effectors, Yki/Yap/Taz can also be regulated by other Ser/Thr kinases (Figure 2). For example, Nuclear Dbf2-related kinases, NDR1 and NDR2, which are structurally related to Lats1/2, can phosphorylate Yap on the same set of sites as Lats1/2 in the intestine epithelium (Zhang L. et al., 2015). MST4, which is closely related to MST1/2, binds and phosphorylates Yap at Thr83 to inhibit Yap nuclear import and activity independent of the canonical Hippo pathway (An et al., 2020). Deletion of MST4 in mice diminished Yap Thr83 phosphorylation, increased Yap activity, and promoted gastric tumorigenesis (An et al., 2020). Furthermore, loss of MST4 and YapThr83 phosphorylation is associated with poor prognosis of human gastric cancer (An et al., 2020). In response to inflammatory cytokine, TAK1 binds and phosphorylates Yap/Taz independent of Lats1/2 to promote Yap/Taz degradation, which alleviates the inhibition of NFκB, leading to the induction of matrix-degrading enzymes and subsequently cartilage degradation during osteoarthritis pathogenesis (Deng et al., 2018). On the other hand, binding of MK5 (also called MAPKAPK5 or PRAK) to Yap stabilizes Yap independent of Lats1/2, which is required for Yap-driven cancer progression (Seo et al., 2019). Two other studies revealed that in response to cellular energy starvation, AMPK can directly phosphorylate Yap on multiple sites including S61 and S94 to inhibit Yap activity at least in part by interfering with Yap–TEAD interaction (Mo et al., 2015; Wang et al., 2015). In addition, AMPK can also indirectly inhibit Yap by activating Lats (Mo et al., 2015). The regulation of Yap by AMPK appears to be evolutionarily conserved as AMPK, and its upstream kinase LKB1 restricts Yki activity in the *Drosophila* larval central nervous system in a manner independent of the Hpo-Wts kinase cascade (Gailite et al., 2015).

A recent study has revealed that the autophagy kinase Atg1/ULK1 acts in parallel to the Hpo-Wts cascade to restrict Yki activity and tissue growth (Tyra et al., 2020). Genetic experiments indicated that gain-of-function of Atg1 and its activator Acinus suppressed tissued overgrowth induced by Yki overexpression, while loss of Atg1 or Acinus increased Yki target gene expression and tissue growth. Biochemical studies demonstrated that Atg1 directly phosphorylated Yki on two Atg1/ULK1 consensus sites S74 and S97 to block its binding to Sd. Atg1-mediated phosphorylation of Yki is independent of Atg13, underscoring an autophagy-independent function of Atg1 in the regulation of Hippo signaling (Tyra et al., 2020). Atg1 is regulated by multiple pathways, including amino acid starvation that activates Atg1 in a Tor-regulated manner (Kim et al., 2011). Consistently, amino acid starvation increased Yki phosphorylation at S74 *in vivo* in a manner depending on Atg1 but independent of Atg13, suggesting that amino acid starvation could restrict Yki activity through activating Atg1. A previous study demonstrated that Tor is required for Yki to access to its target genes even after it enters the nucleus (Parker and Struhl, 2015). As Tor inhibits Atg1 (Kim et al., 2011), one possibility is that in the absence of Tor, Atg1 activity is elevated, leading to increased Yki phosphorylation at S74 and S97 and thus diminished Yki activity. It awaits to be determined whether Yap/Taz is also regulated by Atg1/ULK1 in response to nutrient deficiency in mammalian cells. Because YapS94, which is equivalent to YkiS97, is phosphorylated by AMPK in response to cellular energy starvation (Mo et al., 2015; Wang et al., 2015), phosphorylation at this conserved site could be a general mechanism to regulate Yki/Yap/Taz, which links nutrient starvation to growth inhibition.

The Hippo–Yap and the receptor tyrosine kinase ERBB2 signaling pathways are both required for heart regeneration after injury (D’Uva et al., 2015; Wang et al., 2018). A recent study uncovered a crosstalk between these two pathways in cardiomyocytes (CMs) in a heart failure mouse model (Aharonov et al., 2020). Transient overexpression of an activated form of ERBB2 in CMs induced an epithelial–mesenchymal transition (EMT)-like response to promoted cardiac regeneration (Aharonov et al., 2020). Through a combination of proteome and phospho-proteome analyses coupled with RNA-seq, Yap was identified as a critical mediator downstream of ERBB2 signaling in CMs (Aharonov et al., 2020). ERBB2 overexpression altered the mechanical state of CMs in part by enhancing the interaction of YAP with cytoskeletal and nuclear-envelope components (Aharonov et al., 2020). In addition, ERBB2 signaling promoted Yap phosphorylation at S352 and S274 (S367 and S289 in human Yap) through ERK, which is required for ERBB2 signaling-stimulated CM mitosis during heart regeneration (Aharonov et al., 2020).

## Regulation of Yki/Yap/Taz by Nuclear Kinases

Although most of the regulatory events pertaining the control of Yki/Yap/Taz activity occur in the cytoplasm, a recent study identified a nuclear kinase, PRP4K, as a novel and conserved Hippo pathway component that directly phosphorylates Yki/Yap/Taz and excludes its nuclear localization (Cho et al., 2018). In a genetic modifier screen for genes whose loss of function modified the tissue overgrowth phenotype-caused Yki overexpression, Cho et al. (2018) found that RNAi knockdown of PRP4K enhanced, whereas overexpression of PRP4K, but not its kinase dead form, suppressed the eye overgrowth phenotype caused by eye-specific overexpression of Yki (*GMR* > *Yki*). Further genetic studies indicated that PRP4K acted downstream of Wts but upstream of Yki to regulate Hippo pathway target gene expression and tissue growth. Biochemical studies demonstrated that PRP4K phosphorylates Yki on a subset of Wts sites (S111 and S250), which leads to its nuclear exclusion and reduced interaction with Sd. The function of PRP4K in the Hippo pathway is evolutionarily conserved as PRP4K also acts downstream of Lats1/2 to phosphorylate Yap/Taz on a subset of Lats1/2 sites (excluding the YapS127/TazS89) to restrict their nuclear localization and interaction with TEAD. Hence, phosphorylation of Yki/Yap/Taz regulates their subcellular localization via two distinct mechanisms: phosphorylation of YkiS168/YapS127/TazS89 by Wts/Lats1/2 promotes the binding of Yki/Yap/Taz to 14-3-3, which sequesters Yki/Yap/Taz in the cytoplasm; phosphorylation of Yki/Yap/Taz on other Wts/Lats1/2 sites by Wts/Lats1/2 or PRP4K promotes their nuclear-to-cytoplasmic translocation through a 14-3-3 independent mechanism (Ren et al., 2010). Interestingly, high PRP4K expression correlates good prognosis in triple-negative breast cancer (TNBC) patients, suggesting that PRP4K may function as a tumor suppressor (Cho et al., 2018). Indeed, inactivation of PRP4K in a TNBC cell line MDA-MB-231 promoted cell growth and invasion (Cho et al., 2018). It would be interesting to determine whether and how the action of PRP4K is regulated in development, regeneration, or cancer. Because phosphorylation of Yki/Yap/Taz in the cytoplasm is dynamic, and dephosphorylation by several phosphatases including PP2A and PP1 leads to their nuclear translocation (Liu et al., 2011; Hu et al., 2017; Hein et al., 2019), PRP4K-mediated phosphorylation of Yki/Yap/Taz in the nucleus may provide a fail-safe mechanism to restrict aberrant pathway activity due to unchecked nuclear translocation of these pathway effectors.

In the same genetic modifier screen, Cho et al. (2020) identified the cyclin-dependent kinase 7 (CDK7) as a suppressor of the eye overgrowth phenotype caused by *GMR* > *Yki*. The effect of CDK7 on Yki-driven Hippo pathway target gene expression and tissue growth is independent of its role in cell cycle regulation or Pol II-mediated basal transcription but rather due to its ability to phosphorylate Yki in the nucleus and protect the nuclear Yki from ubiquitin/proteasome-mediated degradation independent of Wts (Cho et al., 2020). Further study demonstrated that CDK7 phosphorylated Yki on S169 and that a phospho-mimetic mutation (YkiS169D) rendered the mutant Yki insensitive to CDK7 inhibition (Cho et al., 2020). CDK7 plays a conserved role in mammalian Hippo pathway and can phosphorylate Yap/Taz on S128/S90 to stabilize the Hippo pathway effectors in the nucleus independent of Lat1/2 (Cho et al., 2020). Pharmacological inhibition of CDK7 by THZ1 rescued the liver overgrowth phenotype caused by MST1 and MST2 double knockout in mice and impeded Yap/Taz-driven tumor cell growth in xenografts (Cho et al., 2020). An independent study revealed a good correlation between CDK7 and Yap protein levels in malignant pleural mesothelioma (MPM) and showed that knockdown of CDK7 in MPM cells reduced Yap level, tumor cell migration and invasion, as well as tumor sphere formation (Miao et al., 2020). Taken together, these studies suggest that CDK7 could be an attractive drug target for Yki/Taz-driven cancers. It is interesting to note that NLK and CDK8 can also phosphorylate Yap at S128 to increase its activity and that phosphorylation of Yap by NLK is induced by osmotic stress (Hong et al., 2017; Moon et al., 2017; Zhou et al., 2018), suggesting that phosphorylation at this site could be regulated by multiple upstream inputs.

## Regulation of Yki/Yap/Taz by Ubiquitination

Lats1/2 phosphorylates Yap/Taz on multiple sites including YapS127/TazS94 and YapS381/TazS311. While phosphorylation of YapS127/TazS94 restricts their nuclear access by promoting their binding to 14-3-3, phosphorylation of YapS381/TazS311 primes further phosphorylation by CK1 on adjacent sites, creating a docking site for the F-box protein β-TRCP (Liu et al., 2010; Zhao et al., 2010), which is a substrate recognition subunit of a family of modular E3 ubiquitin ligases containing SKP1-Cul1-F-box protein (SCF) complexes (Jiang and Struhl, 1998; Spencer et al., 1999). SCF^β –*TRCP*^-mediated ubiquitination targets Yap/Taz for proteasome-mediated degradation (Liu et al., 2010; Zhao et al., 2010). Hence, Lats1/2-mediated phosphorylation of Yap and Taz not only restricts their nuclear localization but also reduces their protein level.

While SCF^β –*TRCP*^-mediated ubiquitination and degradation has not been shown to regulate Yki stability in *Drosophila*, a recent study identified a Cul4-RING E3 ligase (CRL4) complex as an evolutionarily conserved ubiquitin ligase that regulates Yki/Yap/Taz stability in the nucleus (Cho et al., 2020). RNAi-mediated knockdown of Cul4 as well as DCAF12, which serves as a substrate acceptor subunit in the CRL4 complex, promoted tissue growth driven by Yki (Cho et al., 2020). DCAF12 recruits the CRL4^*DCAF*12^ complex to Yki/Yap/Taz, leading to their ubiquitination and proteasome-mediated degradation, whereas CDK7 phosphorylates Yki/Yap/Taz at S169/S128/S90 to inhibit CRL4^*DCAF*12^ recruitment, leading to Yki/Yap/Taz stabilization in the nucleus (Cho et al., 2020). As a consequence, loss of DCAF12 rescued Yki instability and tissue growth defect caused by CDK7 inactivation (Cho et al., 2020). Hence, CDK7 safeguards Yki/Yap/Taz in the nucleus by protecting them from CRL4^*DCAF*12^-mediated ubiquitination and degradation.

Hippo signaling could be regulated by the ubiquitin/proteasome pathway in a context-dependent manner. A recent study uncovered that PARK2, an E3 ubiquitin ligase implicated in Parkinson disease, could regulate Hippo/Yap signaling in esophageal squamous cell carcinoma (ESCC) (Zhou et al., 2020). Immunochemistry study revealed that PARK2 expression was low in human ESCC samples and reversely correlated with Yap expression, and TCGA data analysis indicated that high PARK2 expression correlated with good prognosis in ESCC patients (Zhou et al., 2020). PARK2 KO in ESCC cell lines increased Yap protein level, Hippo target gene expression, cell proliferation and invasion, and tumor progression in xenografts (Zhou et al., 2020). Mechanistically, PARK2 binds Yap and catalyzes its poly-ubiquitination at K90 (Zhou et al., 2020). Hence, PARK2 functions as a tumor suppressor in ESCC by targeting Yap for ubiquitin/proteasome-mediated degradation.

In addition to being targeted for degradation by ubiquitination, Yap can also be regulated by non-proteolytic ubiquitination that is catalyzed by the SCF E3 ubiquitin ligase complex containing SKP2 (SCF^*SKP*2^) (Yao et al., 2018). In HEK293 cells cultured at low density, SKP2 promoted K63-linked polyubiquitination of Yap at K321 and K497, leading to increased Yap-TEAD association, Yap nuclear accumulation, and transcriptional activity (Yao et al., 2018). SKP2-mediated ubiquitination of Yap is reversed by OTUD1, a deubiquitinase that preferentially cleaves K63-linked polyubiquitin chain (Yao et al., 2018). Consistent with their opposing roles in the regulation of Yap, overexpression of SKP2 and knockdown of OTUD1 promoted cancer cell growth *in vitro* (Yao et al., 2018). Furthermore, high SKP2 is associated with poor whereas high OTUD1 with good prognosis in breast cancer patients (Yao et al., 2018). It remains to be determined whether SKP2/OTUD1-mediated Yap regulation plays any role in development, regeneration, and tumorigenesis by *in vivo* study.

A recent study has demonstrated that, in macrophages, IL-1 induces Yap nuclear localization and protein stability by TRAF6-mediated K63-linked poly-ubiquitination of Yap at K252, which disrupts the interaction between Yap and angiomotin (Liu et al., 2020). Macrophage Yap is upregulated in both patients and mouse atherosclerotic lesions, and myeloid-specific overexpression of Yap in mice promoted the development of atherosclerosis, suggesting that interfering of Yap activation could be a therapeutic opportunity for atherosclerosis (Liu et al., 2020).

## Regulation of Yki/Yap/Taz by Deubiquitination

Protein ubiquitination is a reversible process, and a poly-ubiquitin chain on a substrate can be removed by deubiquitinating enzymes (DUBs). An *in vivo* DUB cDNA screen identified OTUB2 as an enhancer of cancer metastasis (Zhang et al., 2019). OTUB2 promoted cancer stemness and metastasis by deubiquitinating and stabilizing Yap/Taz in a manner independent of Lats1/2 (Zhang et al., 2019). Interestingly, OTUB2 is sumoylated on Lys 233, which promotes its association with Yap/Taz via a conserved but previously uncharacterized SUMO-interacting motif (SIM) in Yap/Taz (Zhang et al., 2019). This sumoylation-mediated interaction is essential for Yap/Taz deubiquitination by OTUB2. As a consequence, sumoylation-deficient OTUB2 and SIM-mutated Yap exhibited diminished metastasis-promoting activity (Zhang et al., 2019). Furthermore, OTUB2 sumoylation is stimulated by EGF and oncogenic KRAS, which is essential for EGF and KRAS to stabilize Yap/Taz (Zhang et al., 2019). In breast cancer patients, there is a good correlation between the levels of KRAS and the levels of OTUB2 sumoylation as wells as the levels of Yap/Taz protein expression (Zhang et al., 2019). Hence, OTUB2 sumoylation represents a novel mechanism that links the oncogenic EGFR-RAS pathway to Yap/Taz activation and a potential therapeutic target for cancer treatment. It remains to be determined whether OTUB2-mediated regulation of Yap/Taz is involved in development and tissue regeneration.

In a search for DUBs that could regulate Yap/Taz-mediated transcriptional luciferase reporter, 8XGTIIC, in cultured liver cancer (HepG2) cells, Zhu et al. (2020) found that knockdown of USP10 had the strongest effect on 8XGTIIC expression among the 98 DUBs tested. They found that USP10 interacted with stabilized Yap/Taz by reverting their ubiquitination (Zhu et al., 2020). As a consequence, inactivation of USP10 promoted YAP/TAZ ubiquitination and proteasome-mediated degradation, and inhibited hepatocellular carcinoma cell growth both *in vitro* and in xenografts (Zhu et al., 2020). In hepatocellular carcinoma patient samples as well as in chemical-induced mouse liver cancers, USP10 expression positively correlated with YAP/TAZ abundance, and high USP10 expression correlated with poor prognosis in hepatocellular carcinoma patients (Zhu et al., 2020). Taken together, this study revealed a role of USP10 in liver cancer by promoting Yap/Taz stability and suggested a potential new strategy for therapeutical intervention.

## Regulation of Yap by Methylation

In addition to being regulated by phosphorylation and ubiquitination, Yap/Taz can also be regulated by other posttranslational modifications such as methylation (Figure 2). Two studies revealed that Yap subcellular localization could be regulated by mono-methylation, yet by different lysine methyltransferases (Oudhoff et al., 2013; Fang et al., 2018). Oudhoff et al. (2013) found that knockout of SET-domain-containing lysine methyltransferase 7 (SET7) in mouse intestinal epithelial cells led to increased frequency of cell proliferation per crypt accompanied by increased Yap nuclear localization and Hippo target gene expression. Consistent with this *in vivo* finding, Yap failed to translocate to the cytoplasm in SET7 KO MEFs grown at high density although phosphorylation of Yap by Lats1/2 at S127 was not affected (Oudhoff et al., 2013). Set7 formed a complex with Yap in the cytoplasm in MEFs grown at high density and promoted mono-methylation of Yap at K494 (Oudhoff et al., 2013). Furthermore, YapK494R failed to localize to the cytoplasm in MEFs grown at high density even though it exhibited normal phosphorylation at S127 (Oudhoff et al., 2013). These observations suggest that SET7-mediated mono-methylation Yap at K494 is required for cell density-mediated cytoplasmic localization of Yap, although the underlying mechanism remains unknown.

In contrast to Yap K494 mono-methylation that promotes cytoplasmic retention of Yap, another study found that mono-methylation of Yap at K342 by methyltransferase SET1A promoted nuclear retention of Yap activity by blocking its nuclear export (Fang et al., 2018). Using mass spectrometry analysis and a rabbit polyclonal antibody that specifically recognized a mono-methylated site on Yap, Fang et al. (2018) found that lysophosphatidic acid (LPA) could stimulate Yap mono-methylation at K342 in cancer cell lines. By screening a panel of methyltransferases, the authors identified SET1A as the only methyltransferase that catalyzed Yap K342 methylation in cancer cells (Fang et al., 2018). LPA treatment and low cell density enhanced the interaction between Yap and SET1A and consequently increased Yap K342 mono-methylation (Fang et al., 2018). SET1A-mediated Yap K342 methylation enhanced Yap nuclear localization, Yap-TEAD transcriptional activity, and tumor growth (Fang et al., 2018). Mechanistically, SET1A-mediated Yap K342 methylation inhibited the association between Yap and the nuclear export receptor CRM1 and blocked Yap nuclear export, leading to its nuclear retention (Fang et al., 2018). Interestingly, tissue microarray-base immunohistochemistry study of human lung adenocarcinoma and colorectal cancer (CRC) revealed a good correlation between high SET1A expression with high YAP expression and K342 methylation (Fang et al., 2018). Analysis of TCGA database indicated that SET1A is highly expressed in a number of types of cancer including lung, colon, and breast cancer and that a high SET1A expression is associated with poor outcome in lung and gastric carcinomas (Fang et al., 2018). Taken together, this study suggests that SET1A-mediated Yap methylation may play an important role in tumorigenesis and thus provides an attractive drug target for cancer treatment.

## Regulation of Yap by O-Glcnacylation

O-linked β-N-acetylglucosamine (O-GlcNAc) is a sugar attachment to Ser/Thr hydroxyl moieties on proteins localized in cytoplasm or nucleus, and protein O-GlcNAcylation is regulated by multiple metabolic nutrients including glucose (Slawson et al., 2010). Two independent studies identified Yap O-GlcNAcylation as a mechanism that regulates Hippo pathway outputs in response to altered glucose metabolism (Peng et al., 2017; Zhang et al., 2017a). Both groups found that O-GlcNAc transferase (OGT) interacts with and O-GlcNacylates Yap and that O-GlcNAcylation reduces Yap binding to, and phosphorylation by, Lats1/2, leading to increased Yap activity and Yap-driven tumor growth (Peng et al., 2017; Zhang et al., 2017a). Intriguingly, Peng et al. (2017) found that Yap Thr 241 is the main Yap O-GlcNAcylation site, whereas Zhang et al. (2017a) identified Ser109 as the major site. Mutating either Yap Thr241 or Ser109 to Ala to block O-GlcNAcylation increased the phosphorylation of the Yap mutants by Lats1/2 and consequently reduced their activity (Peng et al., 2017; Zhang et al., 2017a). It is possible that both sites can be O-GlcNAcylated, but the relative contribution of each site may vary depending on cell types. Zhang et al. (2017a) found that OGT is a transcriptional target of Yap–TEAD, uncovering a positive feedback between Yap and global cellular O-GlcNAcylation. Indeed, in a tissue microarray analysis of over 200 liver cancer samples, a statistically significant positive correlation between Yap expression and global O-GlcNAcylation was observed (Peng et al., 2017). Taken together, these studies suggest that Yap O-GlcNAcylation links glucose abundance to Hippo signaling activity and tumorigenesis and could be a potential therapeutic intervention point for cancer treatment.

## Regulation of Yki/Yap/Taz by Protein–Protein Interaction

In addition to posttranslational modifications, Yki/Yap/Taz subcellular localization, stability, and activity can be modulated by interacting proteins (Figure 2). Early studies have demonstrated that binding of Yki/Yap/Taz to 14-3-3 after their phosphorylation by Wts/Lats1/2 or binding of Yap/Taz to Amot promotes cytoplasmic sequestration of these Hippo pathway effectors (Ren et al., 2010; Zhao et al., 2011). A recent study has unraveled a non-canonical role of intraflagellar transport (IFT) complex B proteins (IFT88, IFT55, and IFT20) in the regulation of Hippo/Yap during cardiogenesis independently of primary cilia (Peralta et al., 2020). IFT proteins form a complex with Yap and AMOTL1 to restrict Yap nuclear localization and activity, and this mechanism plays a key role in restricting the formation of the proepicardium and the myocardium in both zebrafish and mouse embryos (Peralta et al., 2020).

Although Yki contains an N-terminal non-canonical nuclear localization signal (NLS) that binds importin α1 to mediate its nuclear import (Wang S. et al., 2016), binding of Yki/Yap/Taz to the Mask family proteins (Mask in *Drosophila* and ANKHD1/2 in mammals) modulates their nuclear import through a canonical NLS in the Mask proteins (Sidor et al., 2019). Another recent study reported that Mastermind-like (MAML) 1 and 2 binds and promotes Yap/Taz nuclear localization and activity depending on MAML NLS (Kim J. et al., 2020). Interestingly, Yap1-MAML2 fusion events leading to constitutive nuclear localization and activation of the fusion proteins were frequently found in a type of benign skin tumor called poroma and its malignant counterpart porocarcinoma (Sekine et al., 2019). Hence, interacting with multiple binding partners regulates nuclear/cytoplasmic localization and activity of the Hippo pathway effectors.

Once in the nucleus, the activity of Yki/Yap/Taz can be further modulated by other interaction partners. Although several studies have revealed that glucose metabolism can regulate Hippo signaling output though AMPK-mediated phosphorylation or OGT-mediated O-GlcNAcylation of Yap, another study showed that increased glucose metabolism and reprogramming toward aerobic glycolysis in cancer cells can upregulate Yap/Taz target gene expression through phosphofructokinase (PFK1), a key enzyme that regulates glycolysis. PFK1 binds TEAD and promotes Yap/Taz interaction with TEAD (Enzo et al., 2015). In addition, a transcriptional signature associated to aerobic glycolysis correlates with elevated YAP/TAZ activity and is predictive of poor prognosis in breast cancer patients (Enzo et al., 2015). Interestingly, the function of PFK1 is conserved in *Drosophila* where it is required for Yki-driven tissue overgrowth (Enzo et al., 2015).

In *Drosophila*, the ecdysone (Ec) receptor coactivator Taiman (Tai) interacts with Yki to enhance Yki-drive tissue growth and intestinal stem cell proliferation (Zhang C. et al., 2015; Wang C. et al., 2016). Interestingly, the Hippo/Ec pathway cooperativity through the formation of Yki–Tai complex drives a distinct pro-growth transcriptional program including germline stem cell factors whose expression is normally suppressed in developing somatic cells (Zhang C. et al., 2015). Another study showed that the transcription factor Shavenbaby (Svb) is expressed in *Drosophila* renal/nephric stem cells and is required for their maintenance during adulthood by physically interacting with Yki to promote the expression of the inhibitor of apoptosis DIAP1 (Bohere et al., 2018).

In mammalian skeletal stem cells (SSCs), the zinc finger transcription factors Snail and Slug promote stem cell proliferation and differentiation through Yap/Taz (Tang et al., 2016). Deletion of Snail/Slug diminished SSC proliferation and blocked osteogenesis both *in vitro* and in mice (Tang et al., 2016). Snail/Slug forms a complex with Yap/Taz to activate a set of Yap/Taz/TEAD target genes that control SSC proliferation, whereas Snail/Slug forms a complex with Taz/Runx2 to promote the expression of Runx2 target genes involved in osteogenesis (Tang et al., 2016). Mechanistically, the extended SNAG domains of Snail/Slug, which recruit chromatin-modifying enzymes critical for transcriptional repression, mediate interactions with the YAP/TAZ WW domains, and these interactions not only stabilizes Yap/Taz by preventing their interactions with Lats1/2 and thus phosphorylation by Lat1/2, but also promotes Yap/Taz transcriptional activity in the nucleus by increasing their promoter occupancy (Tang et al., 2016). Hence, SSCs employ Snail/Slug–YAP/TAZ complexes to control stem cell function. It would be interesting to determine whether similar mechanisms are utilized by other stem cells to regulate their function.

In a proteomic screen, Zhang et al. (2017b) identified the transcription factor TFCP2 as a binding partner of Yap in liver cancer cells. Loss of TFCP2 attenuated, while gain of TFCP2 enhanced, Yap-driven liver growth (Zhang et al., 2017b). Mechanistically, TFCP2 interacts with the WW domain of Yap through a PSY motif, and this interaction enhances Yap binding to TEAD in addition to increasing Yap stability by preventing it from β-TRCP-mediated ubiquitination (Zhang et al., 2017b). TFCP2 and Yap co-regulated a number of Yap–TEAD target genes important in Yap-driven tumorigenesis (Zhang et al., 2017b). Tissue microarray analysis revealed a statistically significant positive correlation between YAP and TFCP2 in liver cancer samples (Zhang et al., 2017b), consistent with the notion that TFCP2 cooperates with Yap to stimulate liver malignancy.

While many Yap/Taz/TEAD-binding proteins promote its activity, others inhibit Yap/Taz activity in the nucleus. For example, Ski, the transforming protein of the avian Sloan–Kettering retrovirus, can inhibit Taz transcriptional activity by binding to TEAD and recruiting the transcriptional co-repressor NCoR1 (Rashidian et al., 2015). Another study showed that TIAM1, a guanine nucleotide exchange factor specific for RAC1, can shuttle between cytoplasm and nucleus, and that nuclear TIAM1 binds TAZ/YAP and blocks its interaction with TEADs, leading to inhibition of TAZ/YAP target genes involved in EMT, cell migration, and invasion (Diamantopoulou et al., 2017). As a consequence, TIAM1 knockdown increased Yap/Taz activity and CRC cell migration and invasion and high nuclear TIAM1 in clinical specimens associates with increased CRC patient survival (Diamantopoulou et al., 2017).

A recent study revealed that the SWI/SNF chromatin remodeling complex is a mechano-regulated inhibitor of Yap/Taz (Chang et al., 2018). The SWI/SNF complex components, such as ARID1A, are frequently inactivated in a wide range of human cancers. By identifying the nuclear factors that interact with YAP/TAZ using chromatin immunoprecipitation followed by mass spectrometry, Chang et al. (2018) identified several components of the SWI/SNF complex in association with Yap/Taz. They further demonstrated that the SWI/SNF complex interacts with Yap/Taz through ARID1A, which blocks the Yap/Taz–TEAD association and hence Yap/Taz transcriptional activity (Chang et al., 2018). As a consequence, loss of SWI/SNF promotes Yap/Taz-driven tissue growth and tumor formation (Chang et al., 2018). Interestingly, the association of Yap/Taz with the SWI/SNF complex is regulated by mechanical cues. At high mechanical stress, nuclear F-actin binds the ARID1A–SWI/SNF complex, thereby preventing its association with Yap/Taz and allowing the formation of Yap/Taz/TEAD complex (Chang et al., 2018). Hence, TEAD competes with the SWI/SNF to bind Yap/Taz, which is favored by high mechanics. This study suggests that oncogenic activation of Yap/Taz not only requires genetic or epigenetic events that increase nuclear Yap/Taz level but also requires genetic or mechanical influence to remove the inhibitory function of the ARID1A–SWI/SNF complex. Intriguingly, another recent study has revealed that ARID1A endows a permissive chromatin state that promotes Yap to access its target genes involved in hepatocyte-to-progenitor conversion during liver injury and regeneration (Li et al., 2019). Hence, the function of ARID1A in Hippo–Yap signaling is complex and context dependent.

## Conclusion

Although many upstream signals regulate Yki/Yap/Taz activity through the Hpo kinase cascade, an increasing number of studies have uncovered other mechanisms that regulate Yki/YAP/Taz subcellular localization, stability, and activity independent of the Hpo kinase cascade. It is highly anticipated that more new mechanisms will be unraveled in the near future by ongoing studies in many labs around the world. These new mechanisms will provide additional layers for pathway regulation, nodes for pathway cross talks, and opportunities for pathway intervention. It is worth noting that many of the mechanisms uncovered so far relied heavily on *in vitro* culture systems, and their physiological relevance needs to be established by genetic studies in model organisms. Other mechanisms such as regulation of Yap methylation by SET1A and regulation of Yap ubiquitination by PARK2 have been derived from studies using cancer cell lines and xenograft models, leaving unclear whether these mechanisms play a role during development and whether they are evolutionarily conserved. Validating these mechanisms using more relevant disease models is important for harnessing these and other mechanisms for therapeutical intervening to treat Yap/Taz-driven cancer and to facilitate tissue repair and regeneration.

## Author Contributions

YC and JJ wrote the manuscript. Both authors contributed to the article and approved the submitted version.

## Conflict of Interest

The authors declare that the research was conducted in the absence of any commercial or financial relationships that could be construed as a potential conflict of interest.

## References

[B1] Aharonov A. Shakked A. Umansky K. B. Savidor A. Genzelinakh A. Kain D. (2020). ERBB2 drives YAP activation and EMT-like processes during cardiac regeneration. *Nat. Cell Biol.* 22 1346–1356. 10.1038/s41556-020-00588-4 33046882

[B2] An L. Nie P. Chen M. Tang Y. Zhang H. Guan J. (2020). MST4 kinase suppresses gastric tumorigenesis by limiting YAP activation via a non-canonical pathway. *J. Exp. Med.* 217:e20191817. 10.1084/jem.20191817 PMC7971137 32271880

[B3] Badouel C. Gardano L. Amin N. Garg A. Rosenfeld R. Le Bihan T. (2009). The FERM-domain protein Expanded regulates Hippo pathway activity via direct interactions with the transcriptional activator Yorkie. *Dev. Cell* 16 411–420. 10.1016/j.devcel.2009.01.010 19289086

[B4] Bae S. J. Ni L. Osinski A. Tomchick D. R. Brautigam C. A. Luo X. (2017). SAV1 promotes Hippo kinase activation through antagonizing the PP2A phosphatase STRIPAK. *eLife* 6:e30278. 10.7554/eLife.30278 PMC5663475 29063833

[B5] Boggiano J. C. Vanderzalm P. J. Fehon R. G. (2011). Tao-1 phosphorylates Hippo/MST kinases to regulate the Hippo-Salvador-Warts tumor suppressor pathway. *Dev. Cell* 21 888–895. 10.1016/j.devcel.2011.08.028 22075147 PMC3217187

[B6] Bohere J. Mancheno-Ferris A. Al Hayek S. Zanet J. Valenti P. Akino K. (2018). Shavenbaby and Yorkie mediate Hippo signaling to protect adult stem cells from apoptosis. *Nat. Commun.* 9:5123. 10.1038/s41467-018-07569-0 PMC6269459 30504772

[B7] Chang L. Azzolin L. Di Biagio D. Zanconato F. Battilana G. Lucon Xiccato R. (2018). The SWI/SNF complex is a mechanoregulated inhibitor of YAP and TAZ. *Nature* 563 265–269. 10.1038/s41586-018-0658-1 30401838 PMC7612964

[B8] Chen R. Xie R. Meng Z. Ma S. Guan K. L. (2019). STRIPAK integrates upstream signals to initiate the Hippo kinase cascade. *Nat. Cell Biol.* 21 1565–1577. 10.1038/s41556-019-0426-y 31792377

[B9] Cho Y. S. Li S. Wang X. Zhu J. Zhuo S. Han Y. (2020). CDK7 regulates organ size and tumor growth by safeguarding the Hippo pathway effector Yki/Yap/Taz in the nucleus. *Genes Dev.* 34 53–71. 10.1101/gad.333146.119 31857346 PMC6938674

[B10] Cho Y. S. Zhu J. Li S. Wang B. Han Y. Jiang J. (2018). Regulation of Yki/Yap subcellular localization and Hpo signaling by a nuclear kinase PRP4K. *Nat. Commun.* 9:1657. 10.1038/s41467-018-04090-2 PMC5916879 29695716

[B11] Chung H. L. Augustine G. J. Choi K. W. (2016). *Drosophila* Schip1 links expanded and Tao-1 to regulate hippo signaling. *Dev. Cell* 36 511–524. 10.1016/j.devcel.2016.02.004 26954546

[B12] Deng Y. Lu J. Li W. Wu A. Zhang X. Tong W. (2018). Reciprocal inhibition of YAP/TAZ and NF-kappaB regulates osteoarthritic cartilage degradation. *Nat. Commun.* 9:4564. 10.1038/s41467-018-07022-2 PMC6212432 30385786

[B13] Diamantopoulou Z. White G. Fadlullah M. Z. H. Dreger M. Pickering K. Maltas J. (2017). TIAM1 Antagonizes TAZ/YAP both in the destruction complex in the cytoplasm and in the nucleus to inhibit invasion of intestinal epithelial cells. *Cancer Cell* 31 621.e6–634.e6. 28416184 10.1016/j.ccell.2017.03.007 PMC5425402

[B14] Dong J. Feldmann G. Huang J. Wu S. Zhang N. Comerford S. A. (2007). Elucidation of a universal size-control mechanism in *Drosophila* and mammals. *Cell* 130 1120–1133. 10.1016/j.cell.2007.07.019 17889654 PMC2666353

[B15] D’Uva G. Aharonov A. Lauriola M. Kain D. Yahalom-Ronen Y. Carvalho S. (2015). ERBB2 triggers mammalian heart regeneration by promoting cardiomyocyte dedifferentiation and proliferation. *Nat. Cell Biol.* 17 627–638. 10.1038/ncb3149 25848746

[B16] Enzo E. Santinon G. Pocaterra A. Aragona M. Bresolin S. Forcato M. (2015). Aerobic glycolysis tunes YAP/TAZ transcriptional activity. *EMBO J.* 34 1349–1370. 10.15252/embj.201490379 25796446 PMC4491996

[B17] Fang L. Teng H. Wang Y. Liao G. Weng L. Li Y. (2018). SET1A-mediated mono-methylation at K342 regulates YAP activation by blocking its nuclear export and promotes tumorigenesis. *Cancer Cell* 34 103.e9–118.e9. 30008322 10.1016/j.ccell.2018.06.002

[B18] Fulford A. Tapon N. Ribeiro P. S. (2018). Upstairs, downstairs: spatial regulation of Hippo signalling. *Curr. Opin. Cell Biol.* 51 22–32. 10.1016/j.ceb.2017.10.006 29154163

[B19] Gailite I. Aerne B. L. Tapon N. (2015). Differential control of Yorkie activity by LKB1/AMPK and the Hippo/Warts cascade in the central nervous system. *Proc. Natl. Acad. Sci. U.S.A.* 112 E5169–E5178. 26324895 10.1073/pnas.1505512112 PMC4577147

[B20] Halder G. Johnson R. L. (2011). Hippo signaling: growth control and beyond. *Development* 138 9–22. 10.1242/dev.045500 21138973 PMC2998162

[B21] Harvey K. F. Pfleger C. M. Hariharan I. K. (2003). The *Drosophila* Mst ortholog, hippo, restricts growth and cell proliferation and promotes apoptosis. *Cell* 114 457–467. 10.1016/s0092-8674(03)00557-9 12941274

[B22] Hein A. L. Brandquist N. D. Ouellette C. Y. Seshacharyulu P. Enke C. A. Ouellette M. M. (2019). PR55alpha regulatory subunit of PP2A inhibits the MOB1/LATS cascade and activates YAP in pancreatic cancer cells. *Oncogenesis* 8:63. 10.1038/s41389-019-0172-9 PMC6817822 31659153

[B23] Hong A. W. Meng Z. Yuan H. X. Plouffe S. W. Moon S. Kim W. (2017). Osmotic stress-induced phosphorylation by NLK at Ser128 activates YAP. *EMBO Rep.* 18 72–86. 10.15252/embr.201642681 27979971 PMC5210094

[B24] Hu J. K. Du W. Shelton S. J. Oldham M. C. DiPersio C. M. Klein O. D. (2017). An FAK-YAP-mTOR signaling axis regulates stem cell-based tissue renewal in mice. *Cell Stem Cell* 21 91.e6–106.e6. 28457749 10.1016/j.stem.2017.03.023 PMC5501749

[B25] Huang J. Wu S. Barrera J. Matthews K. Pan D. (2005). The Hippo signaling pathway coordinately regulates cell proliferation and apoptosis by inactivating Yorkie, the *Drosophila* homolog of YAP. *Cell* 122 421–434. 10.1016/j.cell.2005.06.007 16096061

[B26] Jia J. Zhang W. Wang B. Trinko R. Jiang J. (2003). The *Drosophila* Ste20 family kinase dMST functions as a tumor suppressor by restricting cell proliferation and promoting apoptosis. *Genes Dev.* 17 2514–2519. 10.1101/gad.1134003 14561774 PMC218145

[B27] Jiang J. (2018). Misshapen connects food, mechanosensing, and intestinal growth. *Dev. Cell* 45 417–418. 10.1016/j.devcel.2018.05.004 29787703 PMC6452626

[B28] Jiang J. Struhl G. (1998). Regulation of the Hedgehog and Wingless signalling pathways by the F- box/WD40-repeat protein Slimb. *Nature* 391 493–496. 10.1038/35154 9461217

[B29] Jiao S. Wang H. Shi Z. Dong A. Zhang W. Song X. (2014). A peptide mimicking VGLL4 function acts as a YAP antagonist therapy against gastric cancer. *Cancer Cell* 25 166–180. 10.1016/j.ccr.2014.01.010 24525233

[B30] Kim J. Kundu M. Viollet B. Guan K. L. (2011). AMPK and mTOR regulate autophagy through direct phosphorylation of Ulk1. *Nat. Cell Biol.* 13 132–141. 10.1038/ncb2152 21258367 PMC3987946

[B31] Kim J. Kwon H. Shin Y. K. Song G. Lee T. Kim Y. (2020). MAML1/2 promote YAP/TAZ nuclear localization and tumorigenesis. *Proc. Natl. Acad. Sci. U.S.A.* 117 13529–13540. 10.1073/pnas.1917969117 32482852 PMC7306791

[B32] Kim J. W. Berrios C. Kim M. Schade A. E. Adelmant G. Yeerna H. (2020). STRIPAK directs PP2A activity toward MAP4K4 to promote oncogenic transformation of human cells. *eLife* 9:e53003. 10.7554/eLife.53003 PMC6984821 31913126

[B33] Koo J. H. Guan K. L. (2018). Interplay between YAP/TAZ and Metabolism. *Cell Metab.* 28 196–206. 10.1016/j.cmet.2018.07.010 30089241

[B34] Koontz L. M. Liu-Chittenden Y. Yin F. Zheng Y. Yu J. Huang B. (2013). The Hippo effector Yorkie controls normal tissue growth by antagonizing scalloped-mediated default repression. *Dev. Cell* 25 388–401. 10.1016/j.devcel.2013.04.021 23725764 PMC3705890

[B35] Lai Z. C. Wei X. Shimizu T. Ramos E. Rohrbaugh M. Nikolaidis N. (2005). Control of cell proliferation and apoptosis by mob as tumor suppressor, mats. *Cell* 120 675–685. 10.1016/j.cell.2004.12.036 15766530

[B36] Li P. Silvis M. R. Honaker Y. Lien W. H. Arron S. T. Vasioukhin V. (2016). alphaE-catenin inhibits a Src-YAP1 oncogenic module that couples tyrosine kinases and the effector of Hippo signaling pathway. *Genes Dev.* 30 798–811. 10.1101/gad.274951.115 27013234 PMC4826396

[B37] Li Q. Li S. Mana-Capelli S. Roth Flach R. J. Danai L. V. Amcheslavsky A. (2014). The conserved misshapen-warts-Yorkie pathway acts in enteroblasts to regulate intestinal stem cells in *Drosophila*. *Dev. Cell* 31 291–304. 10.1016/j.devcel.2014.09.012 25453828 PMC4254555

[B38] Li Q. Nirala N. K. Nie Y. Chen H. J. Ostroff G. Mao J. (2018). Ingestion of food particles regulates the mechanosensing misshapen-yorkie pathway in *Drosophila* intestinal growth. *Dev. Cell* 45 433.e6–449.e6. 29754801 10.1016/j.devcel.2018.04.014 PMC7480018

[B39] Li S. Cho Y. S. Yue T. Ip Y. T. Jiang J. (2015). Overlapping functions of the MAP4K family kinases Hppy and Msn in Hippo signaling. *Cell Discov.* 1:15038. 10.1038/celldisc.2015.38 PMC4860773 27462435

[B40] Li W. Yang L. He Q. Hu C. Zhu L. Ma X. (2019). A homeostatic arid1a-dependent permissive chromatin state licenses hepatocyte responsiveness to liver-injury-associated YAP signaling. *Cell Stem Cell* 25 54.e5–68.e5. 31271748 10.1016/j.stem.2019.06.008

[B41] Liu C. Y. Lv X. Li T. Xu Y. Zhou X. Zhao S. (2011). PP1 cooperates with ASPP2 to dephosphorylate and activate TAZ. *J. Biol. Chem.* 286 5558–5566. 10.1074/jbc.m110.194019 21189257 PMC3037669

[B42] Liu C. Y. Zha Z. Y. Zhou X. Zhang H. Huang W. Zhao D. (2010). The hippo tumor pathway promotes TAZ degradation by phosphorylating a phosphodegron and recruiting the SCF{beta}-TrCP E3 ligase. *J. Biol. Chem.* 285 37159–37169. 10.1074/jbc.m110.152942 20858893 PMC2988322

[B43] Liu M. Yan M. Lv H. Wang B. Lv X. Zhang H. (2020). Macrophage K63-linked ubiquitination of YAP promotes its nuclear localization and exacerbates atherosclerosis. *Cell Rep.* 32:107990. 10.1016/j.celrep.2020.107990 32755583

[B44] Meng Z. Moroishi T. Mottier-Pavie V. Plouffe S. W. Hansen C. G. Hong A. W. (2015). MAP4K family kinases act in parallel to MST1/2 to activate LATS1/2 in the Hippo pathway. *Nat. Commun.* 6:8357. 10.1038/ncomms9357 PMC4600732 26437443

[B45] Miao J. Kyoyama H. Liu L. Chan G. Wang Y. Urisman A. (2020). Inhibition of cyclin-dependent kinase 7 down-regulates yes-associated protein expression in mesothelioma cells. *J. Cell Mol. Med.* 24 1087–1098. 10.1111/jcmm.14841 31755214 PMC6933402

[B46] Misra J. R. Irvine K. D. (2018). The hippo signaling network and its biological functions. *Annu. Rev. Genet.* 52 65–87. 10.1146/annurev-genet-120417-031621 30183404 PMC6322405

[B47] Mo J. S. Meng Z. Kim Y. C. Park H. W. Hansen C. G. Kim S. (2015). Cellular energy stress induces AMPK-mediated regulation of YAP and the Hippo pathway. *Nat. Cell Biol.* 17 500–510. 10.1038/ncb3111 25751140 PMC4380774

[B48] Moon S. Kim W. Kim S. Kim Y. Song Y. Bilousov O. (2017). Phosphorylation by NLK inhibits YAP-14-3-3-interactions and induces its nuclear localization. *EMBO Rep.* 18 61–71. 10.15252/embr.201642683 27979972 PMC5210122

[B49] Moya I. M. Halder G. (2018). Hippo-YAP/TAZ signalling in organ regeneration and regenerative medicine. *Nat. Rev. Mol. Cell. Biol.* 20 211–226. 10.1038/s41580-018-0086-y 30546055

[B50] Oh H. Irvine K. D. (2008). In vivo regulation of Yorkie phosphorylation and localization. *Development* 135 1081–1088. 10.1242/dev.015255 18256197 PMC2387210

[B51] Oudhoff M. J. Freeman S. A. Couzens A. L. Antignano F. Kuznetsova E. Min P. H. (2013). Control of the hippo pathway by Set7-dependent methylation of Yap. *Dev. Cell* 26 188–194. 10.1016/j.devcel.2013.05.025 23850191

[B52] Pan D. (2007). Hippo signaling in organ size control. *Genes Dev.* 21 886–897. 10.1101/gad.1536007 17437995

[B53] Pantalacci S. Tapon N. Leopold P. (2003). The salvador partner hippo promotes apoptosis and cell-cycle exit in *Drosophila*. *Nat. Cell Biol.* 5 921–927. 10.1038/ncb1051 14502295

[B54] Parker J. Struhl G. (2015). Scaling the *Drosophila* wing: TOR-dependent target gene access by the hippo pathway transducer yorkie. *PLoS Biol.* 13:e1002274. 10.1371/journal.pbio.1002274 26474042 PMC4608745

[B55] Peng C. Zhu Y. Zhang W. Liao Q. Chen Y. Zhao X. (2017). Regulation of the hippo-YAP pathway by glucose sensor O-GlcNAcylation. *Mol. Cell.* 68 591.e5–604.e5. 29100056 10.1016/j.molcel.2017.10.010

[B56] Peralta M. Ortiz Lopez L. Jerabkova K. Lucchesi T. Vitre B. Han D. (2020). Intraflagellar transport complex B proteins regulate the hippo effector Yap1 during cardiogenesis. *Cell Rep.* 32:107932. 10.1016/j.celrep.2020.107932 32698004

[B57] Poon C. L. Lin J. I. Zhang X. Harvey K. F. (2011). The sterile 20-like kinase Tao-1 controls tissue growth by regulating the Salvador-Warts-Hippo pathway. *Dev. Cell* 21 896–906. 10.1016/j.devcel.2011.09.012 22075148

[B58] Rashidian J. Le Scolan E. Ji X. Zhu Q. Mulvihill M. M. Nomura D. (2015). Ski regulates Hippo and TAZ signaling to suppress breast cancer progression. *Sci. Signal.* 8:ra14. 10.1126/scisignal.2005735 25670202 PMC4457509

[B59] Ren F. Zhang L. Jiang J. (2010). Hippo signaling regulates Yorkie nuclear localization and activity through 14-3-3 dependent and independent mechanisms. *Dev. Biol.* 337 303–312. 10.1016/j.ydbio.2009.10.046 19900439 PMC2812623

[B60] Rosenbluh J. Nijhawan D. Cox A. G. Li X. Neal J. T. Schafer E. J. (2012). beta-Catenin-driven cancers require a YAP1 transcriptional complex for survival and tumorigenesis. *Cell* 151 1457–1473. 10.1016/j.cell.2012.11.026 23245941 PMC3530160

[B61] Sekine S. Kiyono T. Ryo E. Ogawa R. Wakai S. Ichikawa H. (2019). Recurrent YAP1-MAML2 and YAP1-NUTM1 fusions in poroma and porocarcinoma. *J. Clin. Invest.* 129 3827–3832. 10.1172/jci126185 31145701 PMC6715383

[B62] Seo G. Han H. Vargas R. E. Yang B. Li X. Wang W. (2020). MAP4K interactome reveals STRN4 as a key STRIPAK complex component in hippo pathway regulation. *Cell Rep.* 32:107860. 10.1016/j.celrep.2020.107860 32640226 PMC7382313

[B63] Seo J. Kim M. H. Hong H. Cho H. Park S. Kim S. K. (2019). MK5 regulates YAP stability and is a molecular target in YAP-driven cancers. *Cancer Res.* 79 6139–6152. 10.1158/0008-5472.can-19-1339 31578200

[B64] Sidor C. Borreguero-Munoz N. Fletcher G. C. Elbediwy A. Guillermin O. Thompson B. J. (2019). Mask family proteins ANKHD1 and ANKRD17 regulate YAP nuclear import and stability. *eLife* 8:e48601. 10.7554/eLife.48601 PMC6861002 31661072

[B65] Slawson C. Copeland R. J. Hart G. W. (2010). O-GlcNAc signaling: a metabolic link between diabetes and cancer? *Trends Biochem. Sci.* 35 547–555. 10.1016/j.tibs.2010.04.005 20466550 PMC2949529

[B66] Spencer E. Jiang J. Chen Z. J. (1999). Signal-induced ubiquitination of IkappaBalpha by the F-box protein Slimb/beta-TrCP. *Genes Dev.* 13 284–294. 10.1101/gad.13.3.284 9990853 PMC316434

[B67] Tang Y. Fang G. Guo F. Zhang H. Chen X. An L. (2020). Selective inhibition of STRN3-containing PP2A phosphatase restores hippo tumor-suppressor activity in gastric cancer. *Cancer Cell* 38 115.e9–128.e9. 32589942 10.1016/j.ccell.2020.05.019

[B68] Tang Y. Feinberg T. Keller E. T. Li X. Y. Weiss S. J. (2016). Snail/Slug binding interactions with YAP/TAZ control skeletal stem cell self-renewal and differentiation. *Nat. Cell Biol.* 18 917–929. 10.1038/ncb3394 27479603 PMC5007193

[B69] Taniguchi K. Wu L. W. Grivennikov S. I. de Jong P. R. Lian I. Yu F. X. (2015). A gp130-Src-YAP module links inflammation to epithelial regeneration. *Nature* 519 57–62. 10.1038/nature14228 25731159 PMC4447318

[B70] Totaro A. Panciera T. Piccolo S. (2018). YAP/TAZ upstream signals and downstream responses. *Nat. Cell Biol.* 20 888–899. 10.1038/s41556-018-0142-z 30050119 PMC6186418

[B71] Tyra L. K. Nandi N. Tracy C. Kramer H. (2020). Yorkie Growth-Promoting Activity Is Limited by Atg1-mediated phosphorylation. *Dev. Cell* 52 605.e7–616.e7. 32032548 10.1016/j.devcel.2020.01.011 PMC7105283

[B72] Udan R. S. Kango-Singh M. Nolo R. Tao C. Halder G. (2003). Hippo promotes proliferation arrest and apoptosis in the Salvador/Warts pathway. *Nat. Cell Biol.* 5 914–920. 10.1038/ncb1050 14502294

[B73] Wang C. Yin M. X. Wu W. Dong L. Wang S. Lu Y. (2016). Taiman acts as a coactivator of Yorkie in the Hippo pathway to promote tissue growth and intestinal regeneration. *Cell Discov.* 2:16006. 10.1038/celldisc.2016.6 PMC4860958 27462453

[B74] Wang J. Liu S. Heallen T. Martin J. F. (2018). The Hippo pathway in the heart: pivotal roles in development, disease, and regeneration. *Nat. Rev. Cardiol.* 15 672–684. 10.1038/s41569-018-0063-3 30111784

[B75] Wang S. Lu Y. Yin M. X. Wang C. Wu W. Li J. (2016). Importin alpha1 mediates yorkie nuclear import via an N-terminal non-canonical nuclear localization signal. *J. Biol. Chem.* 291 7926–7937. 10.1074/jbc.m115.700823 26887950 PMC4825000

[B76] Wang W. Xiao Z. D. Li X. Aziz K. E. Gan B. Johnson R. L. (2015). AMPK modulates Hippo pathway activity to regulate energy homeostasis. *Nat. Cell Biol.* 17 490–499. 10.1038/ncb3113 25751139 PMC4380807

[B77] Wu S. Huang J. Dong J. Pan D. (2003). hippo encodes a Ste-20 family protein kinase that restricts cell proliferation and promotes apoptosis in conjunction with salvador and warts. *Cell* 114 445–456. 10.1016/s0092-8674(03)00549-x 12941273

[B78] Wu S. Liu Y. Zheng Y. Dong J. Pan D. (2008). The TEAD/TEF family protein Scalloped mediates transcriptional output of the Hippo growth-regulatory pathway. *Dev. Cell* 14 388–398. 10.1016/j.devcel.2008.01.007 18258486

[B79] Yao F. Zhou Z. Kim J. Hang Q. Xiao Z. Ton B. N. (2018). SKP2- and OTUD1-regulated non-proteolytic ubiquitination of YAP promotes YAP nuclear localization and activity. *Nat. Commun.* 9:2269. 10.1038/s41467-018-04620-y PMC5995870 29891922

[B80] Yu F. X. Zhao B. Guan K. L. (2015). Hippo pathway in organ size control, tissue homeostasis, and cancer. *Cell* 163 811–828. 10.1016/j.cell.2015.10.044 26544935 PMC4638384

[B81] Yue T. Tian A. Jiang J. (2012). The cell adhesion molecule echinoid functions as a tumor suppressor and upstream regulator of the Hippo signaling pathway. *Dev. Cell* 22 255–267. 10.1016/j.devcel.2011.12.011 22280890 PMC3288783

[B82] Zanconato F. Cordenonsi M. Piccolo S. (2016). YAP/TAZ at the roots of cancer. *Cancer Cell* 29 783–803. 10.1016/j.ccell.2016.05.005 27300434 PMC6186419

[B83] Zhang C. Robinson B. S. Xu W. Yang L. Yao B. Zhao H. (2015). The ecdysone receptor coactivator Taiman links Yorkie to transcriptional control of germline stem cell factors in somatic tissue. *Dev. Cell* 34 168–180. 10.1016/j.devcel.2015.05.010 26143992 PMC4519380

[B84] Zhang L. Ren F. Zhang Q. Chen Y. Wang B. Jiang J. (2008). The TEAD/TEF family of transcription factor Scalloped mediates Hippo signaling in organ size control. *Dev. Cell* 14 377–387. 10.1016/j.devcel.2008.01.006 18258485 PMC2292673

[B85] Zhang L. Tang F. Terracciano L. Hynx D. Kohler R. Bichet S. (2015). NDR functions as a physiological YAP1 kinase in the intestinal epithelium. *Curr. Biol.* 25 296–305. 10.1016/j.cub.2014.11.054 25601544 PMC4426889

[B86] Zhang L. Yue T. Jiang J. (2009). Hippo signaling pathway and organ size control. *Fly* 3 68–73. 10.4161/fly.3.1.7788 19164949 PMC6404755

[B87] Zhang X. Qiao Y. Wu Q. Chen Y. Zou S. Liu X. (2017a). The essential role of YAP O-GlcNAcylation in high-glucose-stimulated liver tumorigenesis. *Nat. Commun.* 8:15280. 10.1038/ncomms15280 PMC5424161 28474680

[B88] Zhang X. Sun F. Qiao Y. Zheng W. Liu Y. Chen Y. (2017b). TFCP2 is required for YAP-dependent transcription to stimulate liver malignancy. *Cell Rep.* 21 1227–1239. 10.1016/j.celrep.2017.10.017 29091762

[B89] Zhang Z. Du J. Wang S. Shao L. Jin K. Li F. (2019). OTUB2 promotes cancer metastasis via hippo-independent activation of YAP and TAZ. *Mol. Cell.* 73 7.e7–21.e7. 30472188 10.1016/j.molcel.2018.10.030

[B90] Zhao B. Li L. Lu Q. Wang L. H. Liu C. Y. Lei Q. (2011). Angiomotin is a novel Hippo pathway component that inhibits YAP oncoprotein. *Genes Dev.* 25 51–63. 10.1101/gad.2000111 21205866 PMC3012936

[B91] Zhao B. Li L. Tumaneng K. Wang C. Y. Guan K. L. (2010). A coordinated phosphorylation by Lats and CK1 regulates YAP stability through SCF(beta-TRCP). *Genes Dev.* 24 72–85. 10.1101/gad.1843810 20048001 PMC2802193

[B92] Zhao B. Wei X. Li W. Udan R. S. Yang Q. Kim J. (2007). Inactivation of YAP oncoprotein by the Hippo pathway is involved in cell contact inhibition and tissue growth control. *Genes Dev.* 21 2747–2761. 10.1101/gad.1602907 17974916 PMC2045129

[B93] Zhao B. Ye X. Yu J. Li L. Li W. Li S. (2008). TEAD mediates YAP-dependent gene induction and growth control. *Genes Dev.* 22 1962–1971. 10.1101/gad.1664408 18579750 PMC2492741

[B94] Zheng Y. Liu B. Wang L. Lei H. Pulgar Prieto K. D. Pan D. (2017). Homeostatic control of Hpo/MST kinase activity through autophosphorylation-dependent recruitment of the STRIPAK PP2A phosphatase complex. *Cell Rep.* 21 3612–3623. 10.1016/j.celrep.2017.11.076 29262338 PMC5741103

[B95] Zheng Y. Pan D. (2019). The hippo signaling pathway in development and disease. *Dev. Cell* 50 264–282. 10.1016/j.devcel.2019.06.003 31386861 PMC6748048

[B96] Zheng Y. Wang W. Liu B. Deng H. Uster E. Pan D. (2015). Identification of Happyhour/MAP4K as alternative Hpo/Mst-like kinases in the hippo kinase cascade. *Dev. Cell* 34 642–655. 10.1016/j.devcel.2015.08.014 26364751 PMC4589524

[B97] Zhou J. Zeng Y. Cui L. Chen X. Stauffer S. Wang Z. (2018). Zyxin promotes colon cancer tumorigenesis in a mitotic phosphorylation-dependent manner and through CDK8-mediated YAP activation. *Proc. Natl. Acad. Sci. U.S.A.* 115 E6760–E6769. 29967145 10.1073/pnas.1800621115 PMC6055133

[B98] Zhou X. Li Y. Wang W. Wang S. Hou J. Zhang A. (2020). Regulation of Hippo/YAP signaling and esophageal squamous carcinoma progression by an E3 ubiquitin ligase PARK2. *Theranostics* 10 9443–9457. 10.7150/thno.46078 32863938 PMC7449928

[B99] Zhu H. Yan F. Yuan T. Qian M. Zhou T. Dai X. (2020). USP10 promotes proliferation of hepatocellular carcinoma by deubiquitinating and stabilizing YAP/TAZ. *Cancer Res.* 80 2204–2216. 10.1158/0008-5472.can-19-2388 32217697

